# Quality Improvement in Anesthetic Management of Patients with Left Ventricular Assist Device Support Presenting for Non-Cardiac Surgery: A Single Center Experience

**DOI:** 10.3390/jcm13051421

**Published:** 2024-02-29

**Authors:** Dana D Yahav-Shafir, Ascher Jekutiel Gérard Schmelczer, Jonathan Frogel, Ilya Matskovsky, Amir Zabida, Jonathan Eisenberger, Jeffrey A. Morgan

**Affiliations:** 1 Department of Anesthesiology, Sheba Medical Centre, Ramat Gan 5262000, Israelimatskovsky@gmail.com (I.M.);; 2 School of Medicine, Tel Aviv University, Tel Aviv 6139001, Israel; 3 Department of Cardiac Surgery, Leviev Cardiothoracic and Vascular Centre, Sheba Medical Centre, Ramat Gan 5262000, Israel; jonathaneisenberger1@gmail.com

**Keywords:** left ventricular assist device, anesthesia, heart failure

## Abstract

With the growing number of left ventricular assist device (LVAD) recipients requiring non-cardiac surgery and the limited availability of cardiac anesthesiologists, our study reviewed non-cardiac surgeries in HeartMate III patients with LVAD at our institution. We focused on anesthesiologist roles, detailing patient characteristics, anesthetic management, and outcomes and identifying improvement opportunities in this specialized care setting. A retrospective chart review was conducted of all patients with LVAD who underwent non-cardiac surgery at our institution between 2017 and 2022. Patient demographics, surgical characteristics, anesthetic management, and 30-day mortality rates were also assessed. A total of 23 patients were identified, with 17 (73.9%) males and a median age of 61 [53.5, 67.5] years. Cardiac anesthesiologists were present in nine (39.1%) cases. Elective surgeries were more common (73.9%), with intermediate-risk surgeries accounting for 52.2% of all surgeries. General anesthesia was administered to 18 patients (78.3%), with a median duration of 40 [24, 63.5] min. A single patient required reoperation because of bleeding, and two patients (8.7%) experienced 30-day mortality. Despite guidelines lacking detail, involving non-cardiac anesthesiologists in certain cases is essential. Sharing our experience aims to enhance the evolving discourse on non-cardiac surgeries for patients with LVAD, improving their outcomes and safety.

## 1. Introduction

Over the past two decades, the implantation of left ventricular assist devices (LVADs) has gained popularity in Israel and worldwide [1,2]. Since the first recorded successful LVAD implantation by Dr. Michael DeBakey in 1966, these devices have undergone significant evolution, leading to notable improvements in the quality of life and survival rates of patients with end-stage systolic left heart failure [3,4,5].

With accepted indications for LVAD implantation, including bridge-to-recovery, bridge-to-transplant, and destination therapy, the HeartMate III has emerged as the most frequently implanted LVAD in Israel since 2015. Consequently, Sheba Medical Centers’ LVAD team gained significant experience, extending beyond the perioperative setting. Our LVAD team includes both operative and perioperative personnel—Cardiac surgeons, Anesthesiologists, Perfusionists, Intensivists, Nurse practitioners well as other consultants such as—Cardiologists, and Coagulation specialists.

As the prevalence of patients with LVAD needing non-cardiac surgery increases, the necessity for anesthesiologists to have a thorough understanding of the device and its specific perioperative considerations has become evident. Consequently, a critical aspect that demands attention is the decision regarding which type of anesthesiologist should administer anesthesia—cardiac or non-cardiac. This decision is particularly challenging given the scarcity of cardiac anesthesiologists relative to the rising patient population. In response to these challenges and the lack of explicit guidelines, our goal was to conduct an exhaustive review of all non-cardiac surgeries performed on patients with HeartMate III LVADs at our facility, focusing explicitly on the anesthesiologist’s role. Our intention was to convey our insights from managing these cases, delineate their specificities, elaborate on the detailed anesthesia management, and document the outcomes, thereby pinpointing potential areas for enhancement.

## 2. Materials and Methods

We retrospectively reviewed the medical charts of all patients with HeartMate III LVAD who required an anesthesiologist for non-cardiac surgery at our institution from October 2017 to July 2022. Data collected included demographic details, perioperative clinical variables, laboratory results, and anesthetic data. Follow-up assessments were conducted for up to one month post-surgery. All patients presenting for non-cardiac surgical procedures, including those undergoing transcatheter ablation in the electrophysiology suite and under the care of an anesthesiologist with an LVAD at our institution, were included in this study. Patients who underwent bedside procedures with or without sedation were excluded from the study.

To enhance the specificity of our evaluation, we categorized the patients into two groups based on the anesthesia provider (group 1, cardiac anesthesiologist; group 2, non-cardiac anesthesiologist).

This research project was approved by our local bioethics board (IRB approval number: 0192-23-SMC) and followed the Strengthening the Reporting of Observational Studies in Epidemiology (STROBE) Guidelines [6].

### Statistical Analysis

The normality of the data was evaluated using the Shapiro-Wilk test. Patient demographic and clinical characteristics were summarized using standard descriptive statistics stratified by anesthesia provider group. The groups were described using the absolute standardized mean difference. A formal sample size calculation was not employed because we included the entire population of patients with HeartMate III LVAD undergoing non-cardiac procedures. All statistical analyses were implemented using Python Software Foundation, Python Language Reference, version 3.9.12, and the ‘Pandas’ package.

## 3. Results

Patient and procedure characteristics are presented in Table 1.

### 3.1. Demographics

We identified 23 consecutive patients with HeartMate III LVAD who underwent non-cardiac surgical procedures. These procedures were categorized as having minor, intermediate, and major risks (6/23, 5/23, and 12/23, respectively). The median time from LVAD implantation to the procedure was 412 days [98, 874.5]. Among these cases, nine (39.1%) were managed by a cardiac anesthesiologist (group 1), while 14 (60.9%) were managed by a non-cardiac anesthesiologist (group 2). The median age was 61 [53.5, 67.5] years, and 16 patients were males (73.9%). Most procedures (20/23, 86.9%) were performed in the main operating room. Indications for LVAD implantation were equally distributed between ischemic cardiomyopathy (12 patients, 52.2%) and dilated cardiomyopathy (11 patients, 47.8%). General anesthesia was administered to 18 (78.3%) patients, and six (26.1%) were classified as urgent or emergent (Table 1).

### 3.2. Pre-Operative Management of Anti-Coagulation

In this study, of the 23 patients enrolled, 14 were undergoing warfarin therapy at the time of presentation. Among these, four patients’ pre-operative management involved discontinuing warfarin 96 to 36 h prior to surgery without the use of “bridging” therapy with low molecular weight heparin (LMWH). Conversely, five patients ceased warfarin treatment and received “bridging” therapy with LMWH, while three patients proceeded to surgery under warfarin therapy. Notably, none of the patients required the administration of blood products perioperatively. Furthermore, three patients were administered pre-operative vitamin K and prothrombin complex concentrates. Specific details regarding pre-operative anticoagulant status, the International Normalized Ratio (INR) value prior to surgery, and intraoperative blood product therapy are presented in Table 1.

### 3.3. Postoperative Outcomes

Following the surgical procedures, most patients (13 out of 23, 56.5%) were successfully extubated at the end of the case. Four patients (17.4%) were not intubated during their procedures, and two patients (8.7%) had a tracheostomy in place. The remaining four patients (17.4%) were not extubated at the end of the procedure for various clinical reasons. The median Hospital Length of Stay was 5 days with a median of one day in the Cardiac Surgery Intensive Care Unit (ICU).

## 4. Discussion

Our quality improvement project focused on evaluating the anesthetic management of non-cardiac surgeries in patients with HeartMate III LVADs at our institution, emphasizing the crucial role of the anesthesiologist. The growing population of LVAD recipients in need of non-cardiac surgery poses a challenge, particularly with the limited availability of cardiac anesthesiologists [7,8,9].

Notably, our analysis did not reveal any thromboembolic events. The observed mortalities were unrelated to anesthesia, with causes attributed to non-anesthetic surgical interventions and multi-organ failure in the intensive care unit. Importantly, during the procedures involving non-cardiac anesthesiologists, there was no instance where a cardiac anesthesiologist was called for assistance. The absence of clinically significant differences in the management provided by cardiac and non-cardiac anesthesiologists across variables suggests a level of safety in our current practice.

The distinction between emergency and urgent surgeries posed challenges in the medical records, with some cases considered urgent because of after-hour start times. Addressing this variability in recording practices may enhance clarity in future studies.

Our anesthesia department, situated in a tertiary, university-affiliated center, encompasses approximately 100 anesthesiologists, with a specialized team of five focusing on cardiac cases. These cardiac anesthesiologists oversee around 30 LVAD implantations each year. In instances where patients with LVAD undergo non-cardiac procedures, our standard protocol involves the pre-induction placement of an arterial line and a peripheral intravenous catheter, either 18- or 16-gauge. During elective cases, an LVAD nurse aids with the console setup and patient transport, ensuring smooth logistical operations. Our protocol anticipates the potential need for LVAD nurse or cardiac anesthesiologist consultation in instances of LVAD-related questions or complications. The LVAD nurse is available for elective cases, while a cardiac anesthesiologist is on standby for emergencies, ready to assist in the operating room as needed, although we have not yet encountered such a situation. In scenarios where the native left ventricular pulsatility is deemed insufficient, we supplement our routine monitoring with cerebral saturation measurements using near-infrared spectroscopy rather than depending solely on peripheral pulse oximetry probes or non-invasive blood pressure cuffs. Additionally, it is important to note that all patients included in this study were hospitalized for their procedures, with none scheduled as same-day admissions. Furthermore, we did not utilize transesophageal echocardiography for any patient in this cohort. Should the need for one arise, our protocol involves consulting a cardiac anesthesiologist or a cardiologist proficient in echocardiography. In our study, the postoperative management strategy for these patients includes a routine transfer to the Cardiac Surgery ICU for at least one day, regardless of the procedure type.

Currently, standardized protocols for anesthesia delivery, peripheral nerve block, and hemodynamic support choices are lacking. Case audits guided our decisions, emphasizing the maintenance of hemodynamic goals and understanding the unique physiology of patients with LVADs [10,11]. Clear communication and a multidisciplinary approach involving hematologists, electrophysiology technicians, and LVAD nurses are crucial.

A comparison with other case series revealed a novel aspect of our study, as none of the patients were divided based on the attending anesthesiologist. The existing literature primarily reports mortality and major complication outcomes, with rates ranging from 0% to 7.7% and transfusion rates varying from 0% to 42.5% [12,13,14]. Anesthetic agent recommendations in the literature remain scarce [12,15], emphasizing the need for further investigations to establish comprehensive guidelines.

### 4.1. Anesthetic Management for the Patient with LVAD Undergoing Non-Cardiac Surgery

Anesthetic management should begin with a pre-operative evaluation and consider coexisting comorbidities, current drug therapy, functional status or frailty status, presence of chronic or acute infection, and presence of a pacemaker or electronic implantable cardiac device. A multidisciplinary team, including a coagulation specialist, cardiologist, intensivist, and cardiac surgeon, should participate in the pre-operative evaluation as needed. Current recommendations for the reversal of warfarin anti-coagulation in patients with LVAD include treatment with vitamin K antagonists with a target INR of 2–2.5 prior to surgery [9,16,17,18]. Recent evidence suggests that because of the increased hemocompatibility of HeartMate III and the consequent reduction in pump thrombosis events, lower INR values should be targeted to prevent bleeding events [17]. At our institution, patients with LVADs are treated with warfarin in the absence of significant active bleeding. Evidence-based recommendations regarding perioperative anti-coagulation management for LVAD HeartMate III patients are scarce. Thus, treatment decisions should consider the risk of major bleeding during and after surgery, as well as the risk of pump thrombosis events.

The magnitude of the planned procedure, complexity, risk of bleeding, urgency, and patients’ current INR and hemoglobin values will guide the need for blood product availability. The authors propose that, for each procedure, blood samples ought to be dispatched to the blood bank for typing and antibody screening. This step is crucial since many patients have been exposed to homologous blood products, heightening the risk of antibody development. Furthermore, candidates for heart implantation must receive filtered and irradiated packed cells and platelets when necessary.

To provide optimal anesthetic management for LVAD patients for non-cardiac surgery, it is essential to understand the hemodynamic physiology under LVAD rhythm, rate, RV contractility, pre-load, and after-load, and the influence of the lack of neurohormonal auto-regulation and continuous flow on these parameters. Understanding the physiology and influence of the patient’s current medical condition, as well as the planned procedure, will guide the anesthetist in choosing anesthetic agents, blood product therapy, and the use of vasopressors.

In this case series, several agents were chosen for the induction and maintenance of anesthesia (Table 2). The most popular choices for induction were combinations of intravenous fentanyl, midazolam, propofol, and etomidate (14/23); ketamine was used on one occasion. In seven cases, a balanced induction technique with inhalational agents, mainly sevoflurane, supplemented with the aforementioned intravenous agents, was chosen. Maintenance therapy is typically performed using isoflurane or sevoflurane combined with fentanyl. Muscle relaxation was achieved exclusively using rocuronium.

Very few recommendations for anesthetic agents exist in the current literature. Pisansky et al. used fentanyl, propofol, and rocuronium for anesthesia induction in a patient with LVAD presenting for robotic prostatectomy [15]. Morgan et al. reported the use of sevoflurane, isoflurane, and desflurane, along with propofol or etomidate, as induction agents, which were chosen according to the anesthesiologist’s preference [12]. Other agents, including midazolam, fentanyl, succinylcholine, and cisatracurium, have been used without adverse effects [11,14].

Specific protocols are unavailable because as long as hemodynamics are stable, LVAD physiology and patient-related factors are maintained, and any combination of hypnotics, analgesic drugs, and muscle relaxants is acceptable. This observation was strongly supported by our findings. At our institution, we don’t recommend one specific agent over another as long as the abovementioned considerations are judiciously made.

Regarding vasoactive and ionotropic medications, as per our institution’s protocol, we recommend starting a phenylephrine infusion before induction. Vasopressin and norepinephrine were administered less frequently. Two critically ill patients were intubated for emergency procedures with inotropic support using dobutamine and dobutamine plus milrinone. In all elective cases, inotropic support other than phenylephrine and norepinephrine was not required. Upon reflecting on our experience, we can assert that basic vasoactive medications generally suffice for LVAD patient care. However, we advise preparing additional cardiovascular medications, particularly vasopressin, for immediate availability.

All patients with LVAD were monitored with electrocardiogram, pulse oximetry, ETCO2, and temperature when necessary according to the American Society of Anesthesiologists standards, as well as additional NIRS monitoring. Due to LVAD support offering continuous flow, as opposed to pulsatile flow, non-invasive blood pressure measurements using automated cuffs may not always be feasible. Therefore, the Doppler method for blood pressure measurement is recommended [19,20,21]. At our institution, Doppler devices are not readily accessible; hence, we routinely opt for invasive blood pressure measurements. In line with our protocol, a low threshold is advised for arterial line placement, which should be conducted by personnel proficient in ultrasound use to minimize the risk of injury in patients often presenting with vascular pathology and on anti-coagulation therapy. Given the potential inaccuracies of pulse oximetry readings in cases of nonpulsatile flow, we advocate for the placement of NIRS electrodes in all procedures where general anesthesia is administered.

For the majority of minor and moderate procedures, we do not consider the use of a central venous catheter and central venous pressure measurements essential unless necessitated by the patient’s status. Conversely, we advise securing large-bore intravenous access prior to induction, thereby facilitating the safe administration of fluids, blood products, and vasoactive medications.

**Figure 1:**
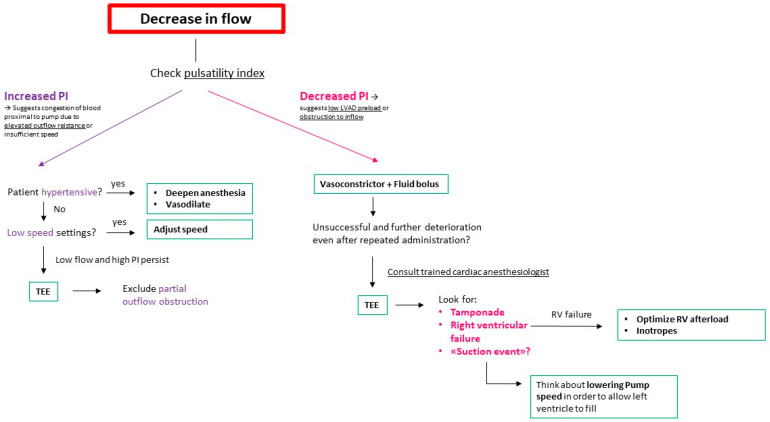
Initial approach schema for decreasing LVAD flow during anesthesia. LVAD: Left Ventricular Assist Device; TEE: Transesophageal Echocardiogram; RV: Right Ventricle; PI: pulsatility index.

In our institution, the majority of these cases are overseen by anesthesiologists who do not specialize in cardiac procedures. While dedicated cardiac anesthesiologists are invariably present during regular working hours, their availability during on-call periods is infrequent. The mechanics of LVAD, including parameters like pump flow, pump speed, pulse index, and pump power, are concepts unfamiliar to providers who do not regularly engage with these patients. Consequently, interpreting changes in flow and pulsatility may present challenges. To address this, we have developed a flowchart algorithm designed to be accessible for practitioners not well-versed in LVAD physiology and mechanics, as shown in Figure 1, furthermore- we offer our “Ten Commandments” for Anesthesia Management for Patients with LVAD. These figures are a result of the consensus within our department, drawing upon our collective experience and best practices rather than direct evidence from the literature.

“Ten Commandments” for Anesthesia Management for Patients with LVAD. NIRS: Near-Infrared Spectroscopy; IV: Intravenous; RBC: Red Blood Cells; TEE: Transesophageal Echocardiography; CIED: Cardiac Implantable Electronic Device; LVAD: Left Ventricular Assist Device.

1. Routine arterial line placement is recommended for all moderate surgeries and above, as well as for all emergency procedures, with a low threshold for minor surgeries.
2. NIRS monitoring should be conducted in conjunction with standard pulse oximetry.
3. Large bore IV access is established pre-induction.
4. Phenylephrine infusion should be administered pre-induction.
5. Vasopressin is prepared in a 20 cc syringe at a concentration of 1 u/cc for both bolus administration and drip.
6. Two units of cross-matched RBC concentrates are available in the OR.
7. TEE is available in the room.
8. A defibrillator is present in the room, regardless of whether the patient has a CIED.
9. The LVAD console is positioned next to the anesthesiologist, ensuring it is readily accessible and visible.
10. A trained cardiac anesthesiologist is informed and available for consultation.

### 4.2. Limitations

Our study had several limitations. First, as this was a non-hypothesis-driven retrospective cohort analysis, we did not perform a formal sample size calculation. A larger sample size is necessary to identify the differences in clinical outcomes related to the provision of anesthesia by a cardiac anesthesiologist. Additionally, retrospective data collection may have introduced bias due to missing data on some variables of interest (e.g., from the device console). Further research with larger sample sizes and more comprehensive data collection strategies is needed to assess the factors influencing outcomes more accurately in patients with LVAD undergoing non-cardiac surgery.

## 5. Conclusions

Overall, based on our limited experience, indicating that the three complicated cases were not anesthesia-related, our findings suggest that our current practice, which allows non-cardiac anesthesiologists to manage some cases, appears safe, particularly in high-volume cardiac centers. We propose that adopting an institutional protocol, maintaining educational sessions, and conducting medical record audits could decrease this knowledge gap and improve the comfort level of non-cardiac anesthesiologists in managing this challenging patient population. By sharing our experience, we aim to contribute to an evolving discussion on the optimal approach to non-cardiac surgeries in patients with LVAD patients, ultimately improving the outcomes and safety of this unique patient population.

## Figures and Tables

**Table 1:** Cardiac vs. Non-Cardiac Anesthesiologist.

		Overall	Group 1 (Yes)	Group 2 (No)	SMD
N		23	9	14	
Age		61.0 [53.5, 67.5]	63.0 [50.0, 69.0]	59.5 [57.0, 66.5]	0.254
Gender	Female	6 (26.1)	3 (33.3)	3 (21.4)	0.269
	Male	17 (73.9)	6 (66.7)	11 (78.6)	
LVAD indication	Dilated cardiomyopathy	11 (47.8)	5 (55.6)	6 (42.9)	0.256
	Ischemic cardiomyopathy	12 (52.2)	4 (44.4)	8 (57.1)	
Classification of Procedure Risk	Minor	6 (26.1)	2 (22.2)	4 (28.6)	0.149
	Intermediate	12 (52.2)	5 (55.6)	7 (50.0)	
	Major	5 (21.7)	2 (22.2)	3 (21.4)	
Urgency	Elective	17 (73.9)	5 (55.6)	12 (85.7)	0.743
	Urgent	4 (17.4)	3 (33.3)	1 (7.1)	
	Emergency	2 (8.7)	1 (11.1)	1 (7.1)	
Location	NORA	3 (13.1)	1 (11.1)	2 (14.3)	0.436
	OR	20 (86.9)	8 (88.9)	12 (85.7)	
Anesthesia type	General	18 (78.3)	8 (88.9)	10 (71.4)	0.449
	Sedation	5 (21.7)	1 (11.1)	4 (28.6)	
Regional Anesthesia		3 (13.0)	1 (11.1)	2 (14.3)	0.095
Time from implantation (days)		412.0 [98.0, 874.5]	670.0 [37.0, 1254.0]	339.5 [115.0, 520.8]	0.381
Procedure Duration (minutes)		40.0 [24.0, 63.5]	40.0 [26.0, 60.0]	40.5 [23.5, 63.8]	−0.162
PRBC		9 (39.1)	4 (44.4)	5 (35.7)	0.179
FFP		4 (17.4)	3 (33.3)	1 (7.1)	0.690
Platelets		1 (4.3)	1 (11.1)		nan
INR		1.6 [1.2, 1.8]	2.0 [1.6, 2.3]	1.4 [1.1, 1.6]	1.348
Thromboembolic Events		0			0.001
Reoperation		1 (4.3)		1 (7.1)	nan
Extubated at the end of the case	Yes	13 (56.5)	6 (66.7)	7 (50.0)	nan
	Not Intubated	4 (17.4)	1 (11.1)	3 (21.4)	
	Tracheostomy	2 (8.7)		2 (14.3)	
	No	4 (17.4)	2 (22.2)	2 (14.3)	
Hospital Length of Stay		5.0 [2.0, 12.0]	5.0 [3.0, 30.0]	4.5 [1.0, 9.0]	0.820
Cardiac Surgery ICU Length of Stay		1.0 [1.0, 1.5]	1.0 [1.0, 4.0]	1.0 [1.0, 1.0]	0.466
30-day mortality		2 (8.7)		2 (14.3)	nan

Values are presented as the median [IQR] or n (%). SMD: standardized mean difference. FFP: fresh frozen plasma; ICU: Intensive Care Unit; INR: international normalized ratio; LVAD: left ventricular assist device; NORA: non-operating room anesthesia; OR: operating room; PRBC: packed red blood cells.

**Table 2:** The procedures encompassed a total of 8 general surgery interventions, including gastrostomy, laparoscopic appendectomy, proctology, laparoscopic inguinal hernia repair, and laparotomy for small bowel obstruction. Additionally, there was one neurosurgery procedure, specifically craniectomy, one otolaryngology procedure involving the excision of tracheal stenosis, two cardiac ablation procedures, three orthopedic interventions such as below-knee amputation, above-knee amputation, and hemiarthroplasty, one vascular surgery procedure comprising thrombectomy, one thoracic surgery procedure involving thoracotomy, two gynecological procedures encompassing hysteroscopy and cervical polypectomy, two gastroenterology procedures, including both upper and lower endoscopy, one urology procedure referred to as RIRS, and finally, one plastic surgery procedure involving the wide excision of a skin tumor.

Patient	Type of Surgery	Midazolam	Opioid	Hypnotic	Ketamine	Rocuronium	Inhalation Agent
1	Gastroscopy and Colonoscopy, active bleeding	Yes	Fentanyl	Propofol	Yes	No	No
2	Craniectomy, hematoma evacuation	Yes	Fentanyl	No	No	Yes	Isoflurane
3	Tracheal stenosis resection and reconstruction	Yes	No	Propofol	No	Yes	Isoflurane
4	Laparoscopic Gastrostomy	Yes	Fentanyl	Propofol	No	No	Sevoflurane
5	Below Knee Amputation	Yes	Fentanyl	No	No	No	No
6	Above Knee Amputation	Yes	Fentanyl	No	No	No	Sevoflurane
7	Thrombectomy lower limb	Yes	Remifentanil	Propofol	No	No	Sevoflurane
8	Thoracotomy, hemothorax	Yes	Fentanyl	No	No	No	Isoflurane
9	Transnasal Polypectomy	No	Fentanyl	Etomidate	No	Yes	Sevoflurane
10	Laparoscopic Appendectomy	Yes	Fentanyl	No	No	Yes	Isoflurane
11	Gastroscopy, polypectomy	Yes	Fentanyl	Both	No	No	Sevoflurane
12	Atrial Flutter Ablation	Yes	Fentanyl	Propofol	No	No	No
13	Hemiarthroplasty, hip	No	Both	Etomidate	No	No	Sevoflurane
14	Hysteroscopy	No	Fentanyl	Etomidate	No	Yes	Sevoflurane
15	Cervical polypectomy	Yes	No	No	No	No	No
16	Open Inguinal Hernia Repair	Yes	Fentanyl	Propofol	No	Yes	Sevoflurane
17	Diagnostic Laparoscopy, small bowel obstruction	Yes	Fentanyl	Etomidate	No	Yes	Isoflurane
18	Anal fistulotomy	No	Fentanyl	Etomidate	No	No	Sevoflurane
19	Tracheal stenosis resection and reconstruction	Yes	Remifentanil	Propofol	No	Yes	No
20	Wide local excision with skin graft	Yes	Fentanyl	Propofol	No	No	Sevoflurane
21	Ureteroscopy, lithotripsy	No	Fentanyl	Propofol	No	No	Sevoflurane
22	Atrial Flutter Ablation	No	Fentanyl	Propofol	No	Yes	Sevoflurane
23	Laparoscopic Inguinal Hernia Repair	Yes	Fentanyl	Propofol	No	Yes	Isoflurane

## Data Availability

The data presented in this study are available on request from the corresponding author.
